# Association of Age at Menarche With Inflammation and Glucose Metabolism Biomarkers in US Adult Women: NHANES 1999-2018

**DOI:** 10.1210/clinem/dgae418

**Published:** 2024-06-24

**Authors:** Maria P Santos, Lydia Bazzano, Owen Carmichael, Sid O’Bryant, Daniel S Hsia, Jiang He, Sylvia H Ley

**Affiliations:** Department of Epidemiology, Tulane University School of Public Health and Tropical Medicine, New Orleans, LA, 70112, USA; Department of Epidemiology, Tulane University School of Public Health and Tropical Medicine, New Orleans, LA, 70112, USA; Pennington Biomedical Research Center, Louisiana State University, Baton Rouge, LA, 70808, USA; Institute for Translational Research, University of North Texas, Fort Worth, TX, 76107, USA; Pennington Biomedical Research Center, Louisiana State University, Baton Rouge, LA, 70808, USA; Department of Epidemiology, Tulane University School of Public Health and Tropical Medicine, New Orleans, LA, 70112, USA; Department of Epidemiology, Tulane University School of Public Health and Tropical Medicine, New Orleans, LA, 70112, USA

**Keywords:** women's health, menarche, inflammation biomarkers, glucose metabolism

## Abstract

**Context:**

Early age at menarche (AAM) is a risk factor for type 2 diabetes later in life, but the pathogenic pathways that confer increased risk remain unknown.

**Objective:**

We examined the associations between AAM and inflammatory and glucose metabolism biomarkers among US adult women who were free of diabetes.

**Methods:**

Using the National Health and Nutrition Examination Survey (NHANES) 1999-2018, 19 228 women over 20 years old who were free of self-reported cancer and diabetes were included in this cross-sectional analysis. AAM was the self-reported age at first menstruation. C-reactive protein (CRP), fasting glucose, fasting insulin, and ferritin levels were measured as biomarkers of inflammation and glucose metabolism in adult blood samples using latex-enhanced nephelometry, enzymatic, and immunoassay methods. Multiple linear regression was used to relate AAM to the biomarkers.

**Results:**

The median age at the time of blood sample collection was 44 years (interquartile range, 33-62). After age adjustment, there was an association between a lower AAM and higher CRP (*P*-trend = .006), fasting glucose (*P*-trend < .0001), fasting insulin (*P*-trend < .0001), and ferritin (*P*-trend < .0001). These remained significant after additional adjustment for demographic, reproductive, lifestyle, and adiposity variables, except for ferritin. Smoking modified the effect of AAM on CRP (*P*-interaction = .014), fasting insulin (*P*-interaction < .001), and fasting glucose (*P*-interaction < .001). In stratified analysis, the observed associations became more pronounced in nonsmokers, while they were attenuated to nonsignificance in active smokers.

**Conclusion:**

Earlier age at menarche is associated with an unfavorable inflammatory and glucose metabolic biomarker profile in a nationally representative sample of adult women free of diabetes, especially among nonsmokers.

Over 500 million people in the world have diabetes, and the prevalence of diabetes is increasing (1, 2). Besides causing its own array of adverse health outcomes (3-5), diabetes also increases the risk for other chronic diseases later in life such as cardiovascular disease, chronic kidney disease, and Alzheimer's disease (6-8). Therefore, early prevention of diabetes has high public health importance. Early age at menarche (AAM), which is frequently used as an indicator of pubertal timing in epidemiologic studies (6), has been identified as an early life risk factor for type 2 diabetes (9-14), although the pathogenic pathways conferring increased risk for girls who experience early AAM remain unknown. Considering the average timing of pubertal age is decreasing in the United States and worldwide (15-17), it is critical to understand the pathway by which early AAM influences the risk of diabetes.

Inflammatory pathways are one pathway through which the risk of diabetes is increased (18). Prior studies of AAM and inflammatory or metabolic markers among middle-aged or older women did not carefully control for the timing of menopause, smoking, or possible interactions between these 2 factors (13, 19-22). For example, international studies that controlled for menopause timing omitted assessment of interactions with smoking, with both such studies reporting nonsignificant associations between AAM and fasting insulin and contradictory associations for fasting glucose (10, 15). US-based studies included only premenopausal women (20) or only postmenopausal women (17), and studies with both pre- and postmenopausal women controlling for menopause timing omitted the assessment of smoking as an effect modifier (22). As a result, these studies yielded contradictory results in terms of the effect of AAM on C-reactive protein (CRP) and glucose metabolism. This is important because menopause is associated with metabolic changes related to adrenal, thyroid, and sex hormone fluctuations (particularly estrogen), which are associated with poor glucose metabolism; therefore, accounting for when in the reproductive lifespan the inflammatory and glucose metabolism measurements were collected is important to better assess the relationship between AAM and these biomarkers (23). Smoking is known to lower estrogen levels in women and could interact with the hypothesized effect of early AAM on inflammation and poorer glucose metabolism (24, 25); however, no studies have assessed smoking as a potential effect modifier of the association between AAM and biomarkers of inflammation and glucose metabolism. Thus, exploring associations between AAM and CRP, fasting insulin, and fasting glucose on a nationally representative sample with a wide age range of both pre- and postmenopausal women to elucidate possible pathways for the increased risk in diabetes in those with earlier AAM is warranted.

The objective of this analysis is to examine the associations of age at menarche with biomarkers of inflammation and glucose metabolism, including CRP, fasting insulin, fasting glucose, and ferritin, among US adult women using the National Health and Nutrition Examination Survey (NHANES) data from 1999-2018.

## Methods

### Study Population

**Figure 1.**
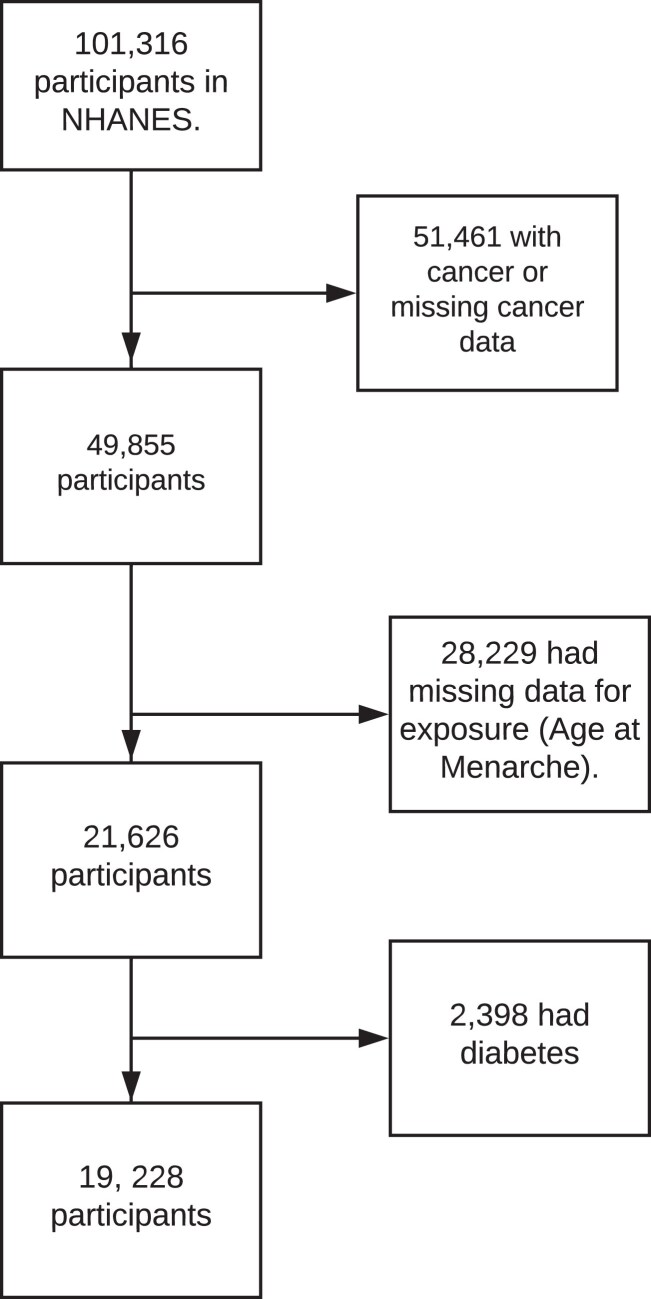
Flowchart of participant inclusion.

This study used data from the NHANES (RRID:SCR_013201) (26) 1999-2000, 2001-2002, 2003-2004, 2005-2006, 2007-2008, 2009-2010, 2011-2012, 2013-2014, 2015-2016, and 2017-2018. The NHANES is a nationally representative cross-sectional survey that collects information among the noninstitutionalized US population (26). The survey employs a multistage probabilistic design to collect a wide range of health information through household interviews and physical examinations (27). In this analysis, we included participants aged 20 years and older who were free of cancer and responded to the reproductive health question asking their age at menarche. As the presence of diabetes introduces a modifying influence on the relationship between age at menarche and the biomarkers currently under investigation, we opted to exclude women who had self-reported diabetes diagnoses (n = 2398), resulting in a total sample size of 19 228 included in the current analysis. In the current analysis, 119 women self-reported taking glucose-lowering medication, and we included them in the present analysis. A flowchart of participant inclusion is available in Fig. 1.

No institutional ethical approval was necessary for this analysis, and research subjects had provided informed consent as part of NHANES participation.

### Ascertainment of Diabetes

All participants answering “Yes” or “Borderline” to the NHANES question asking if they have ever been told by a health practitioner that they had diabetes or sugar diabetes outside of pregnancy were defined as a diabetes case (26). If the participant refused or did not know the answer, the response was counted as missing and excluded from the analysis.

### Assessment of Biomarkers

Laboratory procedures on the collection, processing, and analysis of CRP, fasting glucose, and fasting insulin are available in detail in the NHANES Laboratory Procedures Manual (28). Briefly, specimens were obtained using standard phlebotomy techniques and subsequently frozen at −20°C until laboratory analysis. For fasting glucose, participants were instructed to fast for at least 8 hours before their scheduled examination. A blood sample was then collected using phlebotomy techniques. The blood sample was analyzed in a laboratory using enzymatic or hexokinase methods to measure fasting glucose levels. These methods provided accurate and reliable measurements of glucose concentration in the blood. For fasting insulin, the collected blood sample was analyzed via immunoassay methods. These immunoassay methods utilized antibodies to detect and quantify insulin levels in the blood. For ferritin measurements, the serum was analyzed using immunoassay techniques, such as ELISA. ELISA involved using antibodies that bind specifically to ferritin, allowing its concentration to be quantified. NHANES cycles between 1999 and 2014 quantified CRP by latex-enhanced nephelometry, and NHANES cycles between 2015 and 2018 employed a highly sensitive assay technique to analyze CRP levels, utilizing latex-enhanced nephelometry with a Behring Nephelometer Analyzer System for quantification. For the cycles preceding NHANES 2015-2016, low-sensitivity CRP was converted to high-sensitivity CRP by dividing low-sensitivity CRP by 9.2 (29). CRP was kept as a continuous variable measured in mg/L, fasting glucose was kept as a continuous variable measured in mmol/L, and fasting insulin was kept as a continuous variable measured in pmol/L.

### Assessment of Age at Menarche

Age of menarche was assessed from the question “How old were you when your first menstrual period occurred?” (26) Age of menarche was categorized into 6 groups— ≤ 10, 11, 12, 13, 14, and ≥15 years of age—for main regression analyses.

### Covariates

A number of potential confounders were included in this analysis based on previous literature (14, 16, 25, 30), and availability of data in the NHANES (26): demographic and socioeconomic status including (1) age (continuous), (2) race/ethnicity (non-Hispanic White, non-Hispanic Black, Hispanic, other, or missing), (3) education (<high school graduate, high school graduate, >high school graduate, or missing), (4) poverty income ratio (less than 1 means that the income is less than the poverty threshold, equal to 1 means the income and poverty threshold are the same, and when the ratio is greater than 1, the income is higher than the poverty threshold; <1.0, 1.0-1.99, ≥2.0, or missing), and (5) marital status (married, unmarried, or missing); lifestyle variables such as (6) smoking status (current smoker, former smoker, nonsmoker, or missing), and (7) moderate or vigorous physical activity (yes, no, or missing); and other potential confounding factors such as (8) family history of diabetes (yes, no, or missing), (9) body mass index (BMI) (<25.0, 25.0-29.9, 30.0-34.9, ≥35.0, or missing), (10) parity (0, 1, 2 or more, or missing), menopause (premenopausal, postmenopausal, or missing) and (11) menopause-related hormone therapy (yes, no, or missing). We included all causes of menopause as long as they had 12 consecutive months without a menstrual period, per the NHANES question, as defined by the three established staging systems: Study of Women's Health Across the Nation (SWAN), Stages of Reproductive Aging Workshop (STRAW) and Penn Ovarian Staging Study (PENN-5) (31). All variables had below 5% missing values, except for physical activity (37%) and alcohol consumption (19%). An indicator variable for missingness was used to handle missing data, as indicated in the covariate categorizations.

### Statistical Analysis

Weighted linear regression models were performed to obtain the least square means of the biomarkers by AAM group to investigate the associations between age at menarche and CRP, fasting glucose, fasting insulin, and ferritin. The normality of CRP, fasting glucose, fasting insulin, and ferritin were tested. To account for the skewness within the data, the log-link function was employed in the model to analyze the data. Four sets of models were used, adjusting for previously known risk factors for elevated CRP, fasting insulin, fasting glucose, and ferritin (10, 14, 17, 30-32) including adjusting for age (model 1); additionally adjusting for race/ethnicity, education, parity, menopause status, menopause-related hormone therapy, and family history of diabetes (model 2); further adjusting for smoking status, physical activity, and alcohol (model 3); and further adjusting for BMI (model 4). The missing-indicator method was used to treat missing covariate data. We performed stratified analyses by race/ethnicity and by smoking status due to its antiestrogenic effects controlling for the potential confounders listed for model 4 mentioned earlier (25). We assessed interactions between age at menarche and BMI, cardiovascular disease (CVD), parity, menopause status, hypertension, and family history of diabetes. least square mean estimates, 95% confidence intervals (CIs) and linear P-trends were calculated. For CRP, a U-shape nonlinear term was also tested by adding a quadratic term to the model. Tests for trends were conducted by modeling AAM categories (10-15 years) as continuous variables.

To account for the complex survey design of the NHANES, a 20-year weight was calculated by dividing the original 2-year weight by 10 for each woman and appropriate sample weights were used for 1999-2000 and 2001-2002, according to the NHANES analytic guidelines (27). NHANES participant weights are obtained from the inverse of the final probability of being selected into the sample (27).

All analyses were conducted with statistical analysis software SAS 9.4.

## Results

The median age of the 19 228 women included in the current analysis was 44 years [interquartile range (IQR), 33-62]. The median age at menarche was 13 (IQR, 12-14). Table 1 shows characteristics of the participants according to AAM.

**Table 1.** Weighted characteristics of women without diabetes aged 20 years and older according to age at menarche in NHANES 1999-2018

	Age at menarche (years)					
	<=10	11	12	13	14	>=15
Number of participants, n (%)	1788 (8.27)	2772 (12.82)	5595 (25.87)	5262 (24.33)	2963 (13.70)	3246 (15.01)
Age, mean (SD)	44.26 (16.52)	46.03 (17.57)	47.01 (17.81)	48.44 (17.94)	49.08 (18.12)	51.47 (18.03)
BMI, kg/m^2^, mean (SD)	32.55 (8.29)	30.80 (7.74)	29.83 (7.29)	28.83 (7.21)	28.33 (6.82)	28.04 (6.87)
Race, n (%)						
Non-Hispanic White	606 (33.89)	1148 (41.41)	2399 (42.88)	2457 (46.69)	1274 (43.00)	1166 (35.92)
Hispanic	537 (30.03)	862 (31.10)	1514 (27.06)	1292 (24.55)	863 (29.13)	895 (27.57)
Non-Hispanic Black	520 (29.08)	589 (21.25)	1219 (21.79)	1053 (20.01)	542 (18.29)	766 (23.60)
Other race, including multiracial	125 (6.99)	173 (6.24)	463 (8.28)	460 (8.74)	284 (9.58)	419 (12.91)
Education, n (%)						
< High school	403 (22.55)	653 (23.58)	1275 (22.81)	1304 (24.80)	866 (29.28)	1060 (32.69)
High school	416 (23.28)	601 (21.70)	1286 (23.01)	1198 (22.78)	610 (20.62)	737 (22.73)
> High school	968 (54.17)	1515 (54.71)	3028 (54.18)	2756 (54.42)	1482 (50.10)	1446 (44.59)
Regular menstruation, n (%)	844 (47.20)	1195 (43.11)	2409 (43.06)	2114 (40.17)	1178 (39.76)	1188 (36.60)
Hormone therapy, n (%)	307 (17.17)	556 (20.06)	1035 (18.50)	1055 (20.05)	561 (18.93)	575 (17.71)
Menopause status, n (%)						
Premenopause	931 (52.07)	1359 (49.04)	2734 (48.87)	2408 (45.29)	1342 (45.29)	1330 (40.99)
Postmenopause	857 (47.93)	1412 (50.96)	2861 (51.13)	2854 (54.71)	1621 (54.71)	1915 (59.01)
Family history of diabetes, n (%)	1000 (55.93)	1414 (51.01)	2649 (47.35)	2395 (45.52)	1311 (44.25)	1381 (42.54)
Smoking status, n (%)						
Current smoker	380 (21.25)	516 (18.61)	953 (17.03)	932 (17.71)	476 (16.06)	502 (15.47)
Former smoker	319 (17.84)	545 (19.66)	1019 (18.21)	1045 (19.86)	535 (18.06)	519 (15.99)
Never smoker	1089 (60.91)	1710 (621.69)	3619 (64.68)	3281 (62.35)	1950 (65.81)	2221 (68.42)
Number of pregnancies, n (%)						
0	305 (17.06)	455 (16.46)	849 (15.18)	781 (14.86)	409 (13.83)	412 (12.72)
1	202 (11.32)	347 (12.55)	680 (12.16)	598 (11.38)	315 (10.65)	382 (11.79)
2+	1278 (71.60)	1963 (70.99)	4063 (72.66)	3878 (73.77)	2234 (75.52)	2446 (75.49)
Moderately physically active, n (%)	434 (37.13)	688 (39.65)	1429 (40.96)	1320 (40.49)	768 (41.36)	791 (38.10)
Alcohol consumption in the last 12 months, n (%)	1117 (62.47)	1809 (65.26)	3525 (63.00)	3217 (61.14)	1744 (58.86)	1711 (52.71)
Hypertension, n (%)	306 (17.74)	502 (18.58)	955 (17.56)	983 (19.23)	558 (19.44)	735 (23.44)
CVD	113 (6.32)	164 (5.92)	325 (5.81)	309 (5.87)	195 (6.58)	253 (7.80)

Abbreviations: BMI, body mass index; CVD, cardiovascular disease; NHANES, National Health and Nutrition Examination Survey.

### Assessment of Association Between Age at Menarche and CRP

Earlier AAM was associated with higher CRP (*P* for linear trend <.0001; *P* for nonlinear trend = .003; Table 2) with adjustment for age. With additional adjustment for race, education, parity, menopause, family history of diabetes, alcohol consumption, physical activity, smoking, and BMI (model 4), the association remained significant when testing for a linear trend but the nonlinear trend was nonsignificant (*P* for linear trend .006; *P* for nonlinear trend = .06; Table 2). In this model, interaction terms between AAM and menopause status (*P* = .007) and between AAM and active smoking (*P* = .014) were significant. Associations remained significant with additional adjustment for age at menopause and age at first pregnancy. In sensitivity analysis excluding individuals using glucose-lowering medication (n = 141; 0.7%), the reported association remained significant. Sensitivity analyses were performed on a sample excluding patients with prediabetes to assess the impact of prediabetes; the association between age at menarche and CRP remained significant with adjustment for model 4 (*P* for linear trend = .002).

**Table 2.** Least square means (95% confidence interval) of CRP, fasting insulin, fasting glucose, and ferritin according to age at menarche in population without diabetes

Age at menarche	10 years or less	11 years	12 years	13 years	14 years	15 years or more	*P*-trend
CRP (mg/L)							
n	710	1153	2355	2198	1236	1300	
Model 1	1.42 (1.20, 1.67)	1.08 (0.92, 1.28)	1.05 (0.93, 1.18)	0.80 (0.77, 1.03)	0.84 (0.68, 1.03)	0.91 (0.75, 1.10)	.0057
Model 2	1.58 (1.32, 1.90)	1.31 (1.09, 1.56)	1.26 (1.09, 1.56)	1.10 (0.94, 1.29)	1.02 (0.82, 1.27)	1.08 (0.88, 1.31)	.0003
Model 3	1.01 (0.53, 1.93)	0.83 (0.43, 1.57)	0.82 (0.43, 1.56)	0.69 (0.37, 1.30)	0.61 (0.32, 1.16)	0.71 (0.38, 1.37)	<.0001
Model 4	1.65 (1.03, 2.27)	1.53 (0.93, 2.13)	1.56 (0.97, 2.15)	1.50 (0.91, 2.09)	1.41 (0.81, 2.01)	1.60 (1.00, 2.20)	.006
Fasting insulin (pmol/L)							
n	694	1129	2316	2158	1213	1263	
Model 1	84.85 (80.66, 89.20)	69.19 (66.02, 72.50)	68.11 (65.05, 70.37)	67.27 (65.05, 69.60)	64.42 (61.33, 67.68)	60.69 (57.60, 63.94)	<.0001
Model 2	90.20 (85.26, 95.43)	75.35 (71.47, 79.44)	74.91 (71.90, 78.05)	74.46 (71.40, 77.64)	71.12 (67.34, 75.11)	65.88 (67.34, 75.11)	<.0001
Model 3	94.77 (80.12, 112.10)	80.35 (68.03, 94.70)	80.46 (68.37, 94.70)	79.71 (67.67, 93.90)	75.95 (64.25, 89.78)	70.42 (59.58, 83.24)	<.0001
Model 4	87.50 (67.65, 113.18)	75.30 (64.29, 86.31)	77.98 (67.17, 88.79)	79.81 (68.97, 90.65)	78.09 (67.09, 89.09)	77.29 (66.38, 88.21)	.008
Fasting glucose (mmol/L)							
n	710	1153	2355	2198	1236	1300	
Model 1	5.54 (5.47, 5.61)	5.45 (5.40, 5.51)	5.40 (5.35, 5.42)	5.39 (5.35, 5.42)	5.35 (5.30, 5.40)	5.36 (5.31, 5.42)	<.0001
Model 2	5.67 (5.59, 5.74)	5.59 (5.53, 5.66)	5.54 (5.49, 5.58)	5.53 (5.48, 5.57)	5.48 (5.42, 5.45)	5.48 (5.42, 5.53)	<.0001
Model 3	5.69 (5.49, 5.90)	5.63 (5.44, 5.84)	5.58 (5.39, 5.78)	5.57 (5.38, 5.77)	5.52 (5.33, 5.72)	5.52 (5.33, 5.72)	<.0001
Model 4	5.58 (5.37, 5.79)	5.57 (5.36, 5.57)	5.54 (5.34, 5.73)	5.55 (5.36, 5.75)	5.52 (5.32, 5.72)	5.55 (5.35, 5.75)	.03
Ferritin (ug/L)							
n	933	1417	2841	2715	1487	1497	
Model 1	70.50 (65.76, 75.58)	62.97 (59.37, 66.78)	63.38 (60.84, 66.78)	57.42 (54.91, 60.04)	64.53 (61.09, 68.18)	63.08 (59.74, 66.60)	<.0001
Model 2	80.03 (74.50, 85.99)	74.04 (69.49, 78.90)	74.08 (70.63, 77/70)	67.65 (64.28, 71.20)	76.85 (72.43, 81.54)	73.42 (69.22, 77.68)	.18
Model 3	84.99 (69.75, 100.23)	79.77 (64.81, 94.72)	80.29 (65.60, 94.98)	75.30 (60.55, 90.04)	82.54 (67.56, 97.52)	79.82 (64.96, 94.67)	.26
Model 4	78.05 (62.81, 93.30)	73.92 (58.97, 88.87)	74.23 (59.55, 88.91)	69.64 (54.90, 84.37)	76.97 (62.01, 91.94)	75.12 (60.26, 89.97)	.70

Model 1: adjusted by age, model 2: model 1+ race/ethnicity, education, parity, menopause status, menopause-related hormone therapy, family history; model 3: model 2+ smoking status, physical activity, alcohol; model 4: model 3+ body mass index.

Abbreviation: CRP, C-reactive protein.

In an analysis stratified by menopause status, CRP was 3.23 (95% CI 1.34 to 5.11), 2.76 (95% CI 0.87 to 4.64), 2.57 (95% CI 0.70 to 4.43), 2.56 (95% CI 0.69 to 4.43), 2.36 (95% CI 0.48 to 4.23), and 2.55 (95% CI 0.67 to 4.42) mg/L in premenopausal participants with AAM of 10 years or younger, 11, 12, 13, 14, and 15 years or older, respectively; indicating higher CRP levels with decreasing AAM with a *P*-trend of 0.0002 in premenopausal women. A nonsignificant linear trend was observed in postmenopausal women (Supplementary Table S1a and S1b) (33).

**Figure 2.**
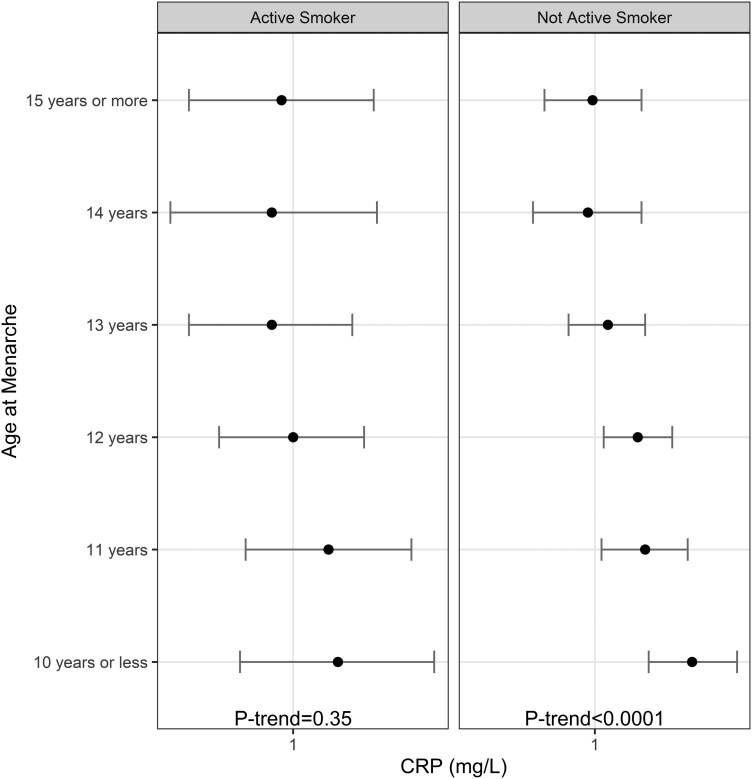
Least square means (95% confidence interval) of CRP according to age at menarche by active smoking status. n for active smoking group = 2645, n for nonactive smoker group = 11976, and *P*-interaction = .014. Abbreviation: CRP, C-reactive protein.

**Figure 3.**
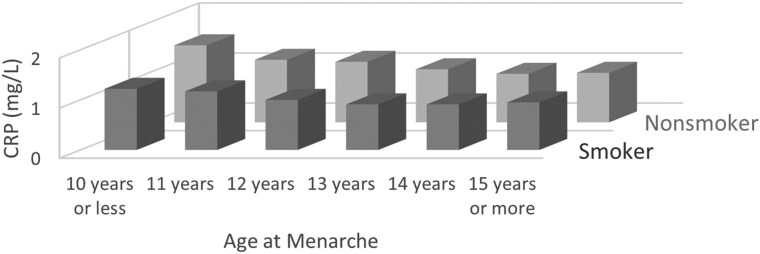
Joint effect of active smoking and age at menarche on least square means of CRP. n for active smoking group = 2645, n for nonactive smoker group = 11976, and *P*-interaction = .014. Abbreviation: CRP, C-reactive protein.

In an analysis stratified by current smoking status, the associations between AAM and CRP remained significant in the nonactive smoking group after multivariate adjustment, but the association was attenuated to nonsignificance in active smokers (Fig. 2). The stepwise decrease of CRP levels observed in nonactive smokers was absent in the active smoker group (Fig. 3).

Interactions between AAM and parity, CVD, hypertension, and family history of diabetes were nonsignificant (*P* for interaction >.05).

Earlier AAM was associated with higher levels of CRP in stratified analyses within White, Hispanic, and Black participant groups, although these trends were nonsignificant (Supplementary Tables S2a-S2c) (33).

### Assessment of Association Between Age at Menarche and Fasting Insulin

Earlier AAM was associated with higher fasting insulin (pmol/L; *P* for trend <.0001; Table 2) with adjustment for age. The association remained significant in fully adjusted model 4 (*P* for trend .008; Table 2). Associations remained significant with additional adjustment for age at menopause and age at first pregnancy. In sensitivity analysis excluding individuals using glucose-lowering medication (n = 141; 0.7%), the reported association remained significant. In this model, interaction terms between AAM and menopause status (*P* = .04) and between AAM and active smoking (*P* = .001) were significant. Sensitivity analyses were performed on a sample excluding patients with prediabetes to assess the impact of prediabetes; the association between age at menarche and fasting insulin remained significant with adjustment for model 4 (*P* for linear trend = .006).

In an analysis stratified by menopause status, fasting insulin was 86.3 (95% CI 54.8 to 117.8), 73.0 (95% CI 41.8 to 104.3), 71.6 (95% CI 40.6 to 102.7), 68.9 (95% CI 37.8 to 99.9), 67.0 (95% CI 35.8 to 98.2), and 62.3 (95% CI 31.1to 93.6) pmol/L in premenopausal participants with AAM of 10 years or younger, 11, 12, 13, 14, and 15 years or older, respectively, indicating higher fasting insulin levels with decreasing AAM with a *P*-trend of <.0001. In the postmenopausal participants, fasting insulin was 92.5 (95% CI 78.7 to 106.3), 79.3 (95% CI 66.1 to 92.5), 81.6 (95% CI 68.8 to 94.3), 82.5 (95% CI 69.7 to 95.3), 79.0 (95% CI 65.9 to 92.2), and 73.6 (95% CI 60.7 to 86.6) pmol/L in participants with AAM of 10 years or younger, 11, 12, 13, 14, and 15 years or older, respectively, indicating higher fasting insulin levels with decreasing AAM with a *P*-trend of <.0001 (Supplementary Table S1a and S1b) (33).

**Figure 4.**
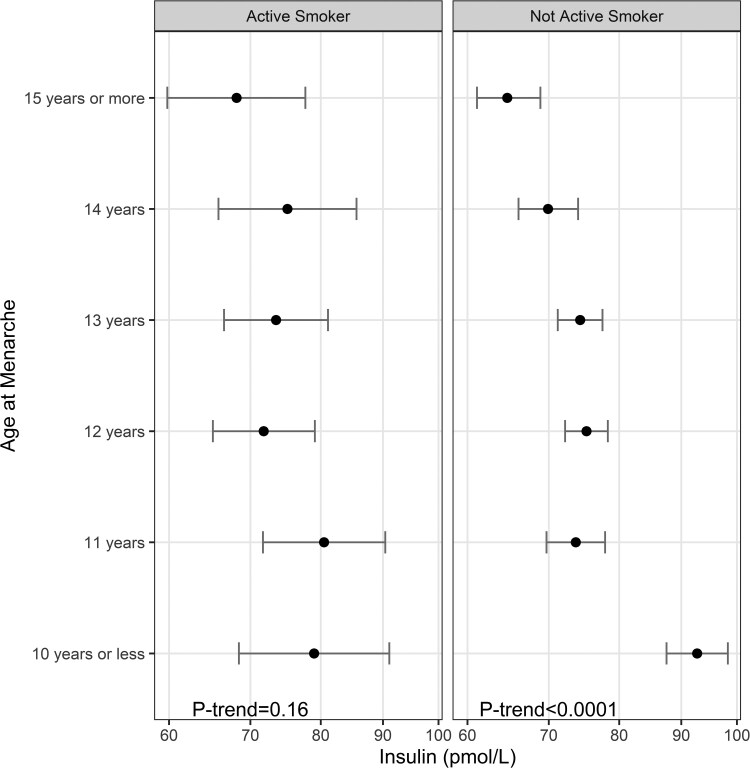
Least-square means (95% confidence interval) of fasting insulin according to age at menarche by active smoking status. n for active smoking group = 1544, n for nonactive smoker group = 7219, and *P*-interaction < .001.

**Figure 5.**
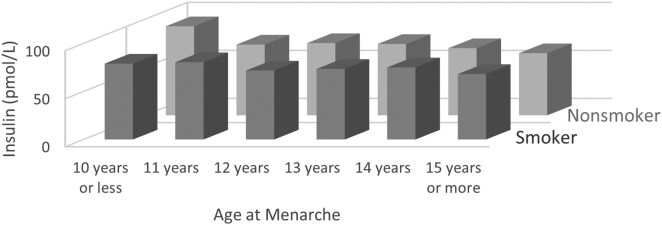
Joint effect of active smoking and age at menarche on least square means of insulin. n for active smoking group = 1544, n for nonactive smoker group = 7219, and *P*-interaction < .001.

In an analysis stratified by current smoking status, the associations between AAM and fasting insulin remained significant in the nonactive smoking group after multivariate adjustment, but the association was attenuated to nonsignificance in active smokers (Fig. 4). The stepwise decrease of fasting insulin levels observed in nonactive smokers was absent in the active smoker group (Fig. 5).

Interactions between age at menarche and parity, CVD, hypertension, and family history of diabetes were nonsignificant (*P* for interaction >.05).

Earlier AAM was associated with higher levels of fasting insulin in stratified analyses within White, Hispanic, and Black participants groups although these trends were only significant among White and Hispanic women (Supplementary Tables S2a-S2c) (33).

Age at menarche was also significantly associated with insulin sensitivity indices such as Homeostatic Model Assessment for Insulin Resistance, Single Point Insulin Sensitivity Estimator, and Quantitative Insulin-sensitivity Check Index. Earlier AAM was associated with higher estimates of Homeostatic Model Assessment for Insulin Resistance (*P* for trend .0019) and lower estimates of Single Point Insulin Sensitivity Estimator (*P* for trend <.0001) and Quantitative Insulin-sensitivity Check Index (*P* for trend .0134) with adjustment for age, race/ethnicity, education, parity, menopause status, menopause-related hormone therapy, family history of diabetes, smoking status, physical activity, alcohol, and BMI (Supplementary Table S3) (33).

### Assessment of Association Between Age at Menarche and Fasting Glucose

Earlier AAM was associated with higher estimates of fasting glucose (*P* for trend <.0001; Table 2) with adjustment for age. The association remained significant in fully adjusted model 4 (*P* for trend .03; Table 2). Associations remained significant with additional adjustment for age at menopause and age at first pregnancy. In this model, interaction terms between AAM and active smoking were significant (*P* < .001). In sensitivity analysis excluding individuals using glucose-lowering medication (n = 141; 0.7%), the reported association remained significant. Sensitivity analyses were performed on a sample excluding patients with prediabetes to assess the impact of prediabetes; the association between age at menarche and fasting glucose remained significant with adjustment for model 4 (*P* for linear trend = .004).

**Figure 6.**
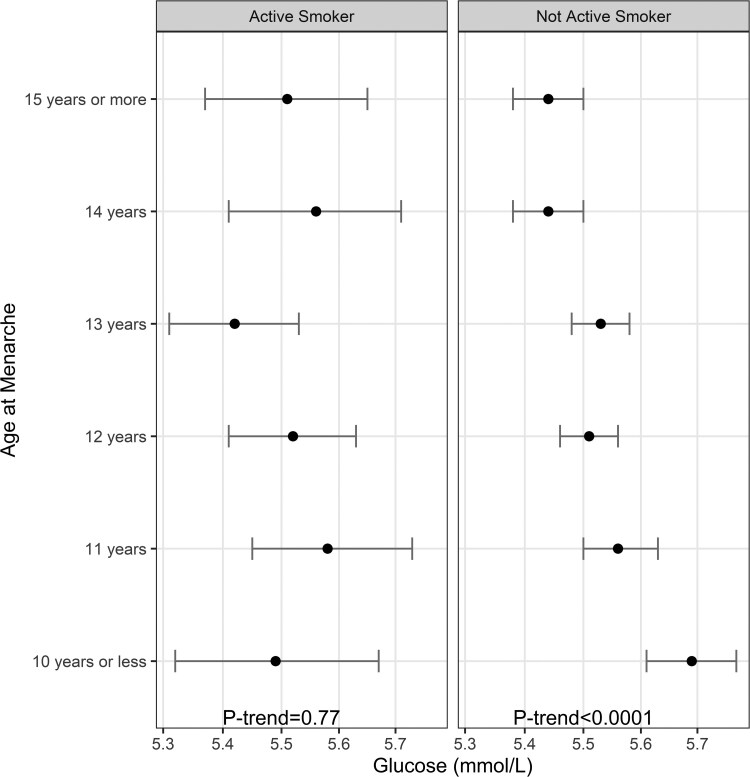
Least-square means (95% confidence interval) of fasting glucose according to age at menarche by active smoking status. n for active smoking group = 1582, n for nonactive smoker group = 7360, and *P*-interaction < .001.

**Figure 7.**
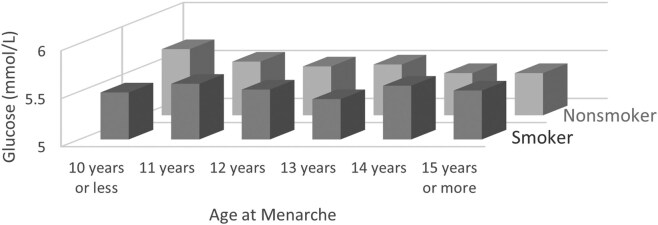
Joint effect of active smoking and age at menarche on least square means of glucose. n for active smoking group = 1582, n for nonactive smoker group = 7360, and *P*-interaction < .001.

In an analysis stratified by current smoking status, linear trends were nonsignificant in the current smoking group, while they remained significant in the nonsmoking group after multivariate adjustment (Fig. 6). The stepwise decrease of fasting glucose levels observed in nonactive smokers was absent in the active smoker group (Fig. 7).

Interactions between AAM and parity, menopause, CVD, hypertension, and family history of diabetes were nonsignificant (*P* for interaction >.05).

Earlier AAM was associated with higher levels of fasting glucose in women with earlier ages at menarche in both pre- and postmenopausal women (Supplementary Table S1a and S1b) (33), and a significant linear trend was observed in both groups. Earlier AAM was associated with higher levels of fasting glucose in stratified analyses within White, Hispanic, and Black participants (Supplementary Tables S2a-S2c) (33), although the trend was nonsignificant in Black participants.

### Assessment of Association of Age at Menarche and Ferritin

Earlier AAM was associated with higher estimates of fasting ferritin (*P* for trend <.0001; Table 2) with adjustment for age. With additional adjustment for race, education, parity, menopause, family history of diabetes, alcohol consumption, physical activity, smoking, and BMI, the association attenuated to nonsignificance (Table 2). Associations remained not significant with additional adjustment for age at menopause and age at first pregnancy. Interactions between AAM and parity, CVD, hypertension, menopause, smoking, and family history of diabetes were nonsignificant (*P* for interaction >.05).

The nonsignificant trends persisted across menopause status (Supplementary Table S1a and S1b) and race and ethnicity subgroup analyses (Supplementary Tables S2a-S2c) (33).

## Discussion

Earlier age at menarche was associated with higher CRP, fasting insulin, and fasting glucose among US women aged median 44 years (IQR, 33-62) after adjusting for age, race/ethnicity, education, parity, menopause status, family history of diabetes, smoking status, physical activity, alcohol, and BMI. Smoking status modified the effect of AAM on CRP, fasting insulin, and fasting glucose, suggesting a weaker effect among active smokers. Our results provide insights for tailored interventions addressing metabolic health in women with earlier AAM.

Similar to our findings, a Polish study also observed an inverse association between AAM and CRP; however, the sample only included women who were premenopausal and failed to control for active smoking (34). The Women's Ischemia Syndrome Evaluation study also reported a significant inverse relationship but in a sample of women with suspected myocardial ischemia, limiting generalizability, and the study did not assess possible interactions between smoking and menopause (35). The US Biocycle study, a Brazilian study, and a Japanese study found nonsignificant results; however, these might be due to being underpowered due to relatively small sample sizes (13, 20, 36). As for the association between AAM and glucose metabolism, a Korean study (19) also found that earlier AAM was associated with increased fasting insulin but not with fasting glucose in contrast to our results, where we found significant associations for both; this could be due to the differing categorizations of AAM in our samples. In the Korean study, AAM was categorized in only 3 groups early (<13 years), average (13-16 years), and late (>16 years). However, the average age of onset of menstruation is between 11 and 12 years old and declining (17), exposing the need for updated earlier age exposure group categories to be able to disentangle dose-response associations that the present study provides. The Bogalusa Heart Study also found significant inverse relationships between AAM and fasting glucose but was limited to a sample of only premenopausal women (37), unlike our analyses where we were able to explore and observe an association in both pre- and postmenopausal women. To our knowledge, the current analysis is the first to assess the relationship between AAM and CRP and glucose metabolism in a nationally representative sample thoroughly examining both pre- and postmenopausal women and active smoking status at the blood collection.

An early onset of menstruation may be a potential early indicator of the development of cardiometabolic diseases in women (38). Studies have shown that endogenous sex hormones play a crucial role in the pathogenesis of diabetes and that elevated levels of SHBG, bioavailable testosterone, and estradiol are associated with insulin resistance and glucose levels independent of adiposity (39, 40). High levels of estradiol have been shown to inhibit insulin signaling and induce cellular insulin resistance, which could explain the inverse association between early menarche and higher fasting glucose and fasting insulin in the study (41). While there is evidence that higher estrogen favors insulin sensitivity, studies show this may only be within a specific physiological concentration range (42). The longer cumulative exposure to elevated endogenous sex hormones stemming from an early age at menarche could represent such elevated levels that surpass the protective threshold (42). Studies have shown that estrogen and testosterone modify the inflammatory response by influencing cytokine expression in human macrophage cells, and hormone therapy in postmenopausal women has been associated with elevated CRP levels (43, 44). Furthermore, the role of smoking as a modifier of that AAM-CRP relationship supports a hypothesized role of increased estrogen in the observed effects of earlier AAM, as smoking is antiestrogenic and indeed attenuates the effect of an early AAM (24, 25).

Nevertheless, our study has some limitations. The interpretation of study findings should take into consideration that fasting insulin and glucose vary within each individual across premenopause, perimenopause, or postmenopause epochs, but this study lacked longitudinal measurements from each individual that covered all 3 of the epochs. Furthermore, there may be selection bias in NHANES, but we have minimized any errors of representation resulting from sample location characteristics and nonresponse by using enhanced weighting adjustments (45). While reverse causality is unlikely to be an issue because menarche preceded the biomarker samples, the cross-sectional nature of the study design precludes the establishment of causality. The cross-sectional evidence presented in this study suggests the need for further research using a longitudinal design. Moreover, a considerable proportion of the sample of women was excluded from the analysis as they lacked age at menarche data, possibly causing selection bias in our findings. Additionally, there is a possibility of misclassification of age at menarche since it was recalled; however, a study comparing data recorded prospectively with self-reports in middle age found no significant difference between self-reported age at menarche and prospective measurement (46). If this type of measurement error exists, it would result in an underestimation of the association.

## Conclusion

In this nationally representative, racially, and ethnically diverse US population, women with earlier age at menarche had higher systemic inflammation and poorer glucose metabolism. These findings suggest the effect of age at menarche on heightened diabetes risk may be due to its effect on inflammation and glucose metabolism. Further investigations are warranted to prevent the progression of inflammation and glucose intolerance among girls who experience earlier AAM and, therefore, who are at higher risk for diabetes.

## Data Availability

Original data generated and analyzed during this study are included in this published article or in the data repositories listed in “References.”
